# Genetic and environmental risk factors for dementia in African adults: A systematic review

**DOI:** 10.1002/alz.70220

**Published:** 2025-04-28

**Authors:** David Mawutor Donkor, Esther Marfo, Ansumana Bockarie, Edward Jenner Tettevi, Maxwell Hubert Antwi, John Dogah, George Nkrumah Osei, David Larbi Simpong

**Affiliations:** ^1^ Department of Medical Laboratory Science School of Allied Health Sciences University of Cape Coast Cape Coast Ghana; ^2^ Department of Internal Medicine and Therapeutics School of Medical Sciences University of Cape Coast Cape Coast Ghana; ^3^ Department of Biochemistry Cell and Molecular Biology School of Biological Science University of Ghana Accra Ghana; ^4^ Department of Medical Laboratory Science Faculty of Health Sciences Koforidua Technical University Koforidua Ghana

**Keywords:** African population, dementia, environment, genetic, risk factors

## Abstract

Dementia, a leading cause of global mortality, disproportionately impacts sub‐Saharan Africans due to complex genetic and environmental interactions. This systematic review evaluated dementia risk factors among sub‐Saharan Africans, identifying significant genetic and environmental influences using the Preferred Reporting Items for Systematic Reviews and Meta‐Analyses (PRISMA) guidelines. The ATP‐binding cassette subfamily A member 7 (*ABCA7*) gene, linked to dementia in African Americans, and unique genetic variants like those in A‐kinase anchor protein 9 (*AKAP9*) and cytidine deaminase (*CDA*) genes, emerge as potential contributors. Conversely, apolipoprotein E (*APOE*) ε4 shows lesser impact in older sub‐Saharan Africans. Environmental findings highlight that exposure to air pollution, including nitrogen dioxide and particulate matter increases the likelihood of dementia. These findings highlight the role of genetic and environmental diversity in shaping dementia risk profiles. Strategies such as training health‐care professionals, enhancing funding for research, combating stigma through awareness campaigns, and fostering global collaborations are vital to ensure African representation in dementia studies. These efforts aim to improve the knowledge of dementia tailored to sub‐Saharan Africa's needs.

**Highlights:**

- The ATP‐binding cassette subfamily A member 7 (*ABCA7*) gene is strongly associated with dementia risk, particularly in African American populations.
- Apolipoprotein E (*APOE*) ε4, a well‐established risk factor for Alzheimer's disease in Western populations, has a lesser impact in older sub‐Saharan Africans, suggesting unique genetic–environment interactions.
- Exposure to air pollutants, such as nitrogen dioxide and particulate matter, significantly increases dementia risk.
- The development of dementia in sub‐Saharan Africans is influenced by complex interactions between genetic predispositions and environmental exposures, emphasizing the need for tailored prevention strategies.

## 1 INTRODUCTION

Dementia is the seventh leading cause of global mortality,^1^ affecting > 55 million people worldwide, a number projected to triple to > 150 million by 2050.^1^, ^2^ Dementia is an umbrella term for neurodegenerative disorders causing progressive cognitive decline, impacting memory, language, problem solving, and daily functioning.^1^ It is not a normal part of aging but results from brain‐affecting illnesses. Alzheimer's disease (AD), the most common form, accounts for 60% to 70% of global cases.^3^ The rising global dementia burden, largely due to increased life expectancy, necessitates an understanding of contributing genetic, environmental, and lifestyle factors. By 2050, global dementia prevalence is projected to exceed 150 million, with a significant proportion in low‐ and middle‐income countries, including sub‐Saharan Africa.^4^

Region‐specific studies in Africa are essential. The unique genetic diversity and environmental challenges in sub‐Saharan Africans which can affect the expression and impact of specific genes necessitate targeted research to identify distinct risk factors and develop effective prevention and management strategies tailored to these communities​. Potential protective genetic variants or differing gene–environment interactions unique to African ancestry with regard to dementia risk have been reported.^5^

In Ghana, dementia prevalence is estimated to be growing due to an aging population.^6^ However, the genetic landscape of dementia in Ghana and other African nations remains underexplored. Ghana's diverse ethnic composition makes understanding these risk factors crucial for identifying potential unique susceptibilities. Dementia, particularly AD, involves complex interactions between genetic and environmental factors, leading to amyloid beta (Aβ) plaque and tau neurofibrillary tangle accumulation in the brain, causing neuronal damage and cognitive decline.^7^ Africans face increased dementia risk due to multidimensional poverty, encompassing deprivations in education, health, and employment. Psychosocial distress further contributes, particularly among women.^8^ The link between environmental factors and dementia is increasingly recognized, with studies highlighting contributing pollutants.^9^, ^10^, ^11^, ^12^ Air pollution, particularly nitrogen dioxide (NO_2_) and particulate matter (PM), significantly correlates with increased dementia risk. Exposure to toxic industrial emissions has also been linked to higher dementia prevalence, especially vascular dementia.^13^ While environmental risk factors such as high temperatures contribute to dementia risk, genetic predisposition also plays a role. Key genes identified as significant contributors include apolipoprotein E (*APOE*) ε4, ATP‐binding cassette subfamily A member 7 (*ABCA7*), amyloid precursor protein (*APP*), presenilin 1 (*PSEN1*), presenilin 2 (*PSEN2*), and triggering receptor expressed on myeloid cells 2 (*TREM2*).^14^ *TREM2* facilitates Aβ phagocytosis and modulates lipid metabolism;^15^ *TREM2* alterations, especially loss‐of‐function mutations, are linked to higher AD and early‐onset dementia risk.^16^, ^17^ *APP* is vital for synaptic transmission and plasticity, enhancing long‐term potentiation and counteracting Aβ neurotoxicity,^18^, ^19^ modulating synaptic transmission, plasticity, and calcium equilibrium.^20^, ^21^ *APP* also plays a critical role in development and provides neuroprotective benefits.^19^  *PSEN1* and *PSEN2* are essential components of the γ‐secretase complex, which cleaves *APP*, leading to Aβ production. Mutations in *APP*, *PSEN1*, and *PSEN2* directly affect amyloid precursor protein processing, increasing Aβ production, a hallmark of AD.^16^

Most dementia research has focused on populations of Western ancestry, while African‐ancestry populations remain significantly understudied, including in genetic research. One study has identified significant dementia risk loci in individuals of African origin, including associations near *APOE*.^22^ This highlights the importance of examining historically understudied populations, particularly in Africa, to address disparities in dementia research.^22^ Genes such as *ABCA7* and *TREM2*, strongly associated with AD in African American populations, require further investigation within African contexts.^23^, ^24^

This underrepresentation of African populations in dementia‐related environmental and genetic research has created a significant knowledge gap.^25^ This lack of data hinders the development of targeted interventions and contributes to health disparities, as findings from elsewhere may not be directly applicable to Africans.^26^, ^27^ While substantial research has established key genetic risk factors for dementia in Western‐origin populations, the applicability of these risk factors to African populations remains poorly understood.^25^, ^28^, ^29^ For example, while *APOE* ε4 is a major AD risk factor in Western populations, its impact may be less pronounced in African populations, suggesting potentially unique genetic and environmental interactions.

This systematic review provides a focused understanding of genetic and environmental factors associated with dementia in African populations and examines potential differences in genetic risk profiles, contributing to a more comprehensive understanding of dementia in Africa.[Fig alz70220-fig-0001]

## 2 METHODOLOGY

This systematic review was conducted according to PRISMA (Preferred Reporting Items for Systematic Reviews and Meta‐Analyses) guidelines to ensure transparency and rigor. The protocol was registered with PROSPERO (registration ID: CRD42024618290). This review included studies focusing on African adults (≥ 18 years) who were either at risk of or diagnosed with dementia. The studies examined genetic risk factors, such as *APOE* ε4, *ABCA7*, and A‐kinase anchor protein 9 (*AKAP9*), as well as environmental exposures like air pollution and socioeconomic factors. Comparisons included studies analyzing dementia risk in African populations versus non‐African populations or within different African cohorts. The primary outcomes assessed were the prevalence, incidence, and risk associations of genetic and environmental factors with dementia. Studies were excluded if they focused exclusively on non‐African populations; adolescent populations; or if they were review articles, opinion pieces, or editorials without original research data. Non‐African populations were excluded because the primary focus of this review was to assess genetic risk factors in African populations and their differences from Western populations, and including non‐African studies could introduce confounding variables. Similarly, studies on adolescent populations were excluded because dementia predominantly affects older adults, and research on younger populations would not be relevant, as early‐onset dementia is rare and often linked to distinct genetic syndromes rather than late‐onset neurodegenerative diseases. Additionally, review articles, opinion pieces, or editorials without original research data were excluded to ensure the review was based on primary data, as including secondary sources could lead to redundancy or bias in the analysis. The exclusion of studies published in non‐English languages was due to these practical considerations. First, language barriers can hinder the accurate interpretation and critical appraisal of studies, potentially introducing bias or misinterpretation. Additionally, the review team had limited resources for translating non‐English texts, leading to a focus on English‐language studies to ensure consistency and reliability in the analysis. A comprehensive literature search was conducted in PubMed, the Cochrane Library, and Google Scholar, covering studies published from database inception to December 2024. Keywords and Boolean operators were used to capture relevant articles, supplemented by a review of gray literature. Gray literature was examined by searching various sources beyond traditional academic databases, which included government reports, conference proceedings, theses, and possibly unpublished studies to ensure comprehensive coverage of available information. The gray literature search was conducted using databases such as Google Scholar and specific institutional repositories, focusing on reports that might not have been published in peer‐reviewed journals. This approach aimed to capture a broader spectrum of data relevant to dementia risk factors in African populations, ensuring that findings were not limited by publication bias or the availability of research in conventional academic sources.

**FIGURE 1 alz70220-fig-0001:**
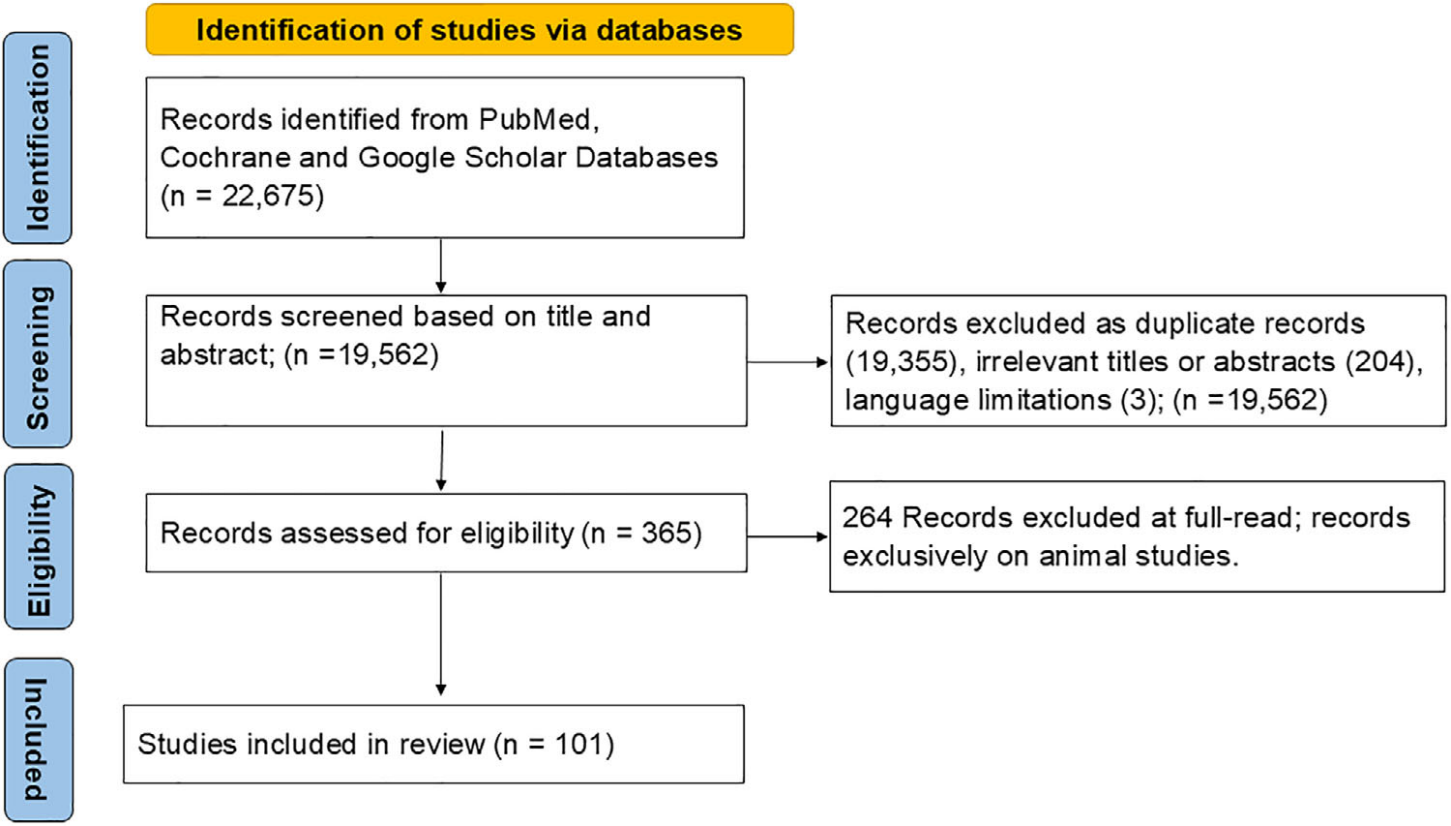
The illustration below is PRISMA flow diagram of studies’ screening and selection. PRISMA, Preferred Reporting Items for Systematic Reviews and Meta‐Analyses.

Detailed search strategies are provided in the supporting information. Two reviewers independently screened titles, abstracts, and full texts, resolving discrepancies through discussion or consultation with a third reviewer. The selection process was documented in a PRISMA flow diagram (Figure 1), detailing reasons for exclusion at each stage. A standardized data extraction form was used to collect study characteristics (e.g., author, year, location, study design), population demographics, risk factors, and outcome measures. Two reviewers independently extracted data, with discrepancies resolved by a third reviewer. To manage the references identified during the systematic review, all retrieved articles were imported into EndNote (version 21.4.18113). The software was used to organize references, facilitate deduplication, and streamline the screening process by categorizing studies into folders based on their eligibility status (included, excluded). EndNote's advanced features, such as automatic full‐text retrieval, were used to annotate, track, and manage decisions made at each stage of the review, ensuring a transparent and reproducible workflow. Risk of bias was assessed using validated tools: the Newcastle–Ottawa Scale (NOS) for cohort and case–control studies, and the Q‐Genie tool for genetic association studies. Two reviewers independently conducted these assessments, resolving disagreements by consensus. A narrative synthesis was performed to summarize findings due to anticipated heterogeneity precluding quantitative pooling (Table 1). As this review relied exclusively on published, publicly available data, no formal ethical approval was required.

**TABLE 1:** Representative characteristics of included genetic & environmental studies.

	Study	Study design	Sample size	Population representation	Genotyping method	Findings
1	Kunkle, 2021	GWAS	*n* = 2784 cases, *n* = 5222 controls	African American	High‐quality GWAS array; *APOE*, *ABCA7* loci identified	*APOE* ε4 was identified as a major risk factor in African Americans, but its effect size was significantly smaller compared to non‐Hispanic Whites.
2	Sherva, 2023	GWAS	*n* = 4012 cases, *n* = 18,435 controls	African descent cohort	GWAS with SNP imputation	The study identified significant associations with dementia risk in an African descent cohort, including notable findings in the *APOE* region
3	Logue, 2018	Targeted sequencing	(*n* = 489 cases, *n* = 472 controls)	African Americans	High‐quality sequencing (*ABCA7*, *AKAP9*)	A rare 11 base‐pair deletion in *ABCA7* was identified in African Americans, increasing AD risk by 2.42‐fold, a much stronger effect than in European cohorts
4	Chen, 2010	Cross‐sectional	East African elderly (*n* = 100)	East African elderly	*APOE* genotyping via PCR	*APOE* ε4 frequency (≈ 30%) was high but showed no significant association with AD
5	Marca‐Ysabel, 2021	Cross‐sectional	79 AD cases and 128 cognitively healthy controls	Admixed population (African/European)	Illumina Global Screening Array v2.0	
6	Reitz, 2014	Case–control	1968 cases, 3928 controls	African Americans	GWAS	*ABCA7* mutations have been linked to a higher risk of AD in African American and Black populations
7	N'songo, 2017	Case–control	340 controls and 198 AD cases	African American	WES	Two *ABCA7* variants, rs3764647, and rs3752239, demonstrated nominal significance in their association with AD risk in a cohort of 538 African American individuals. This highlights the gene's risk of dementia among African ancestry.
8	Naslavsky, 2022	Whole genome sequencing	*Post mortem* sample of 400 individuals	Admixed population (Africans/non‐African)	*APOE* genotyping	Among *APOE* ε4 carriers, the association between AFR proportion and cognitive outcomes (Clinical Dementia Rating Sum of Boxes) disappeared, indicating that the impact of *APOE* genotype on AD neuropathology and cognitive function is significantly influenced by ancestry, with non‐European *APOE* backgrounds showing lower neuropsychiatric burden but worse cognitive outcomes compared to European *APOE* backgrounds.
9	Cukier, 2016	Case–control	1003 cases, 1003 controls	African Americans	WES	
10	Kannan, 2022	Case–control	4012 ADRD cases and 18,435 controls	African ancestry	GWAS	Identified significant risk loci for dementia in African‐ancestry individuals
11	Rajabli et al., 2018	GWAS	1766 African Americans and 220 Puerto Ricans with AD	African ancestry	Genome‐wide array	Ancestry‐specific factors influence ApoE e4 risk in populations. African background reduces Alzheimer risk compared to European background
12	Hui, 1996	Community‐based study of African Americans	268 subjects with *APOE* genotype data	African ancestry	NA	*APOE* ε4 linked to poor dementia screening performance
13	Murrell, 2006	Population‐based longitudinal study	1822 African American subjects	African American	Genotyping of *APOE*	*APOE* ε4 genotype increases AD risk in African Americans
14	Hendrie, 2014	Longitudinal study	1871 African Americans & 2200 Yoruba	Admixed population (African Americans & Yoruba)	*APOE* genotyping	*APOE* ε4 allele and AD seemed to be weaker in Yoruba individuals than in African American cohorts
15	Karch, 2012	Case–control	Autopsy‐confirmed AD (*N* = 73), normal control (*N* = 39) brains	NA	Large‐scale GWAS	Genes such as *ABCA7* were associated with cognitive decline and disease progression in AD
16	Gallagher, 2017	GWAS	7051 samples	NA	GWAS	Variants linked to disease risk of dementia are associated solely with elevated expression of the TMEM106B gene at 7p21. Co‐localization analyses suggest a shared causal variant that connects disease association with TMEM106B expression in human brain. Moreover, heightened TMEM106B levels correlate with increased abnormal lysosomal phenotypes and cytotoxicity in both immortalized cell lines and neurons.
17	Fehér, 2010	Genetic association studies	Multiple studies with varying sample sizes	NA	PCR	Identified associations between various polymorphisms (*APOE*, *BACE1*) and AD, contributing to understanding AD pathogenesis. The *APOE* ε4 allele is the strongest genetic risk factor for sporadic AD, linked to increased amyloid plaque accumulation
18	Matyi, 2023	Population‐based cohort	880 participants (61.3% female, mean age 82.0 years) with dementia	NA	NA	Suggests sex‐dependent effects in dementia progression, possibly due to hormonal interactions with BDNF signaling
19	Stepler, 2022	NA	NA	NA	Biochemical/biophysical studies of *ABCA7* variants	*ABCA7* gene linked to higher AD risk in African Americans
20	Raj, 2016	Longitudinal cohorts	3964 older African Americans	African Americans	GWAS	*ABCA7* and MS4A loci influence cognitive decline in African Americans
21	Bertholim‐Nasciben, 2024	WGS	Frozen brain or peripheral blood samples from 38 individuals	African ancestry	Long read whole genome sequencing (LRWGS)	The A allele of rs10423769, found in African populations, significantly reduces the risk of AD in *APOE* ε4 carriers by ≈ 75%
22	Vasquez, 2022	WGS	1850 African American individuals with AD and 4331 African American controls; 63 Ibadan (Nigerian) AD individuals and 648 Ibadan controls	Admixed	WGS	Lower *APOE* ε2 risk for AD in African ancestry populations
23	Rajabli et al., 2022	WGS	1850 AD, 4331 cognitively intact individuals	African ancestry	WGS, SNP imputation	African ancestry shows lower AD risk compared to European populations
24	Osuntokun, 1995	Community‐based sampling method	56 subjects (39 controls, 17 dementia patients)	Africans	*APOE* genotyping	No association between *APOE* ε4 allele and AD in Nigerians
25	Akinyemi, 2024	Case–control	91 AD cases, 97 controls	Africans	Genome‐wide genotyping	*APOE* ε4 allele significantly associated with AD risk
26	Chaar, 2022	NA	NA	Older African American	NA	*ABCA7* gene linked to cognitive function in older African Americans
27	Sheikh, 2024	Longitudinal study	838 participants aged 60 and older	African ancestry	Genotyping	The *ABCA7*‐80 genotype has been linked to decreased cognitive performance in tasks assessing working memory, indicating its role as a predictive biomarker for early cognitive decline
28	DeRosa, 2023	NA	NA	African ancestry	NA	The *ABCA7* gene's risk effect is notably stronger in African Americans, with specific genetic variations like a frameshift deletion being significantly associated with AD
29	El Kadmiri et al., 2014a	Clinical interviews and genetic sequencing	25 patients (17 sporadic, 8 familial cases)	Africans	Genetic sequencing	Novel mutations in *APP* exons 16 and 17 have been identified in Moroccan patients, suggesting a genetic predisposition to AD
30	N'Songo, 2017	WES and independent cohort analysis	238 African Americans for discovery study	African ancestry	WES	Identified six rare missense variants in *PSEN1* and *PSEN2* among African Americans, which were present in AD cases but absent in controls, suggesting a potential link to increased risk
31	Rippon, 2003	Genetic sequencing of affected and unaffected family members	Two affected brothers from one family	African ancestry	Genetic sequencing	First *PSEN1* mutation documented in African Americans. M139 V mutation linked to atypical AD symptoms.
32	Liu, 2024	Comparison to known PSEN1 homologous variants	74 PSEN2 variants expressed in HEK293 cells	NA	NA	Aβ production correlates with age at symptom onset (AAO). Homologous *PSEN1* variants show similar Aβ production patterns
33	Budak, 2024	Longitudinal study	175 older, cognitively unimpaired African Americans.	African ancestry	Genotyping	*ABCA7*‐80 carriers have lower medial temporal lobe network flexibility than *APOE* ε4 carriers
34	Sheikh, 2024	Longitudinal study	838 participants aged 60 and older	African ancestry	Genotyping	*ABCA7*‐80 high‐risk genotype predicts working memory decline. Associated with decreased cognitive performance and increased depression
35	Cukier et al., 2024	NA	NA	African ancestry	NA	*ABCA7* gene linked to AD risk
36	Nam, 2024	Isogenic iPSC lines from three African American individuals	12 individuals for scRNAseq analysis	African ancestry	CRISPR‐based genome editing in iPSC lines	The research identified a 44‐base pair deletion in *ABCA7* significantly associated with AD in African Americans, suggesting that this African‐specific mutation may influence lipid metabolism and contribute to increased dementia risk in this population
37	Davis, 2022	Self‐identified ethnic/racial groups	4026 cognitively normal participants	African ancestry	Longitudinal data from the Health and Retirement Study evaluated	Compelling evidence indicated that genetic variants of the lipid transport protein ABCA7 are more strongly associated with AD in African Americans, particularly the *ABCA7* variant (T319A), which may contribute to dementia risk by reducing phosphoinositol bisphosphate (PIP2) levels
38	Sinha, 2023	Matched case–control design from a larger study	114 cognitively healthy older African Americans	African ancestry	Genotyping for *ABCA7* risk allele in participants	*ABCA7* rs115550680 is a significant genetic risk factor for late‐onset AD in African Americans, influencing cognitive decline. The interplay between *ABCA7* genotype and sleep quality may further exacerbate cognitive health disparities in this population
39	Berg, 2019	Matched case–control design based on genotype	100 healthy African Americans	NA	*ABCA7* rs3764650 genotype	*ABCA7* risk variants, particularly rs115550680, are linked to higher AD risk in African Americans. However, rs3764650 does not show a direct association with AD, suggesting indirect risk through interactions with lifestyle factors like aerobic fitness
40	Sinha, 2019	Matched case–control design based on age and education	36 healthy older African Americans	African ancestry	Standardized neuropsychological assessments for cognitive functioning evaluation	*ABCA7* risk variants significantly increase the odds of AD in African Americans, with an odds ratio of 1.79. This genetic risk factor is associated with cognitive impairments, particularly in generalization, despite no differences in standardized cognitive assessments.
41	Kurup, 2022	Case–control	2913 cases, 5802 controls	African ancestry	Single‐variant association analysis	The paper identified *ABCA7* as a novel susceptibility locus for Alzheimer disease in African Americans, suggesting its involvement in dementia risk. However, it does not specifically address the relationship between *ABCA7* and dementia risk in Africans
42	Kiianitsa, 2024	NA	NA	African ancestry	Full‐size *TREM2* splicing reporter assay in cell lines. qRT/RT‐PCR and western blots for quantification	TREM2 variants, such as R62H and T96K, are more common in African populations and are associated with splicing defects that may contribute to AD risk
43	Jin et al., 2015	Case–control	906 late‐onset AD cases, 2487 controls	African ancestry	Exonic sequencing	*TREM2* coding variants, particularly p.L211P and p.W191X, are associated with increased risk for late‐onset AD in African Americans, highlighting the need for further investigation into ethnic‐specific genetic factors influencing dementia risk in diverse populations
44	Shafi, 2021	NA	NA	NA	NA	*TREM2* genetic variations linked to neurodegenerative dementia pathogenesis. AD accounts for 50% to 70% of late‐life dementias
45	Karsak, 2020	WES	WES study of a family	NA	WES study of a family	Identified rare *TREM2* variant linked to familial dementia.
46	Logue, 2014	WES	422 cases and 394 controls in discovery cohort	African ancestry	WES and genotyping in cohorts	Two rare *AKAP9* variants have been associated with AD, showing significant odds ratios in African American cohorts
47	Ikezu et al., 2018	NA	11 African American subjects with mutations, 17 without	African ancestry	*APOE* genotype	*AKAP9* mutations increase tau phosphorylation in lymphoblastoid cell lines. No effect on amyloidogenic *APP* processing pathway observed
48	Bertholim‐Nasciben, 2024	Short and long read sequencing	NA	African ancestry	Short and long read sequencing	The A allele of rs10423769, located 2 Mb from *APOE* ε4 was prevalent in African populations, suggesting an ancient origin and a unique haplotype that interacts with *APOE* ε4 to mitigate its effects
49	Guen, 2021	Next generation sequencing	11,790 individuals of African ancestry	African ancestry	Next generation sequencing and microarray data	The paper highlighted that the *APOE* ε4 allele increases AD risk, particularly in African ancestry individuals. It emphasizes the need for ancestry‐specific studies to understand genetic variants' mechanisms and their implications for dementia risk in diverse populations
50	Vanderlip, 2024	NA	NA	NA	NA	*APOE* ε4 increases amyloid deposition and episodic memory decline
51	Sadhukhan, 2024	Whole exome analysis	29 clinically diagnosed AD cases	NA	Whole exome analysis performed	*ABCA7* gene is identified as significant in familial AD cases, with five pathogenic variants found. Its mutations correlate with earlier onset and are linked to specific phenotypes, suggesting a crucial role in the disease's pathogenesis and mechanisms
52	Gialama, 2024	Conducted searches on PubMed, Google Scholar, and Scopus. Applied inclusion and exclusion criteria for study selection	NA	NA	NA	*ABCA7* genetic polymorphisms contribute to AD development, showing racial disparities in risk. African American and Asian populations exhibit higher susceptibility due to specific variants, indicating *ABCA7*'s significant role in the pathogenesis of dementia
53	Snyder, 2023	WGS	Three individuals with *ABCA7* mutations	NA	Genetic testing through WGS	*ABCA7* is an AD risk gene linked to amyloid pathology. Loss of function mutations can lead to behavioral variant frontotemporal dementia, as demonstrated in cases without amyloid pathology, suggesting its involvement in neurodegenerative disease through lipid transport and immune responses
54	Nam, 2024	Isogenic iPSC lines from three African American individuals	12 individuals for scRNAseq analysis	NA	Isogenic iPSC lines from three African American individuals	The *ABCA7* gene, associated with AD, produces a truncated protein due to a frameshift deletion. This alteration may disrupt lipid metabolism, contributing to dementia pathogenesis, particularly in African Americans, as shown through isogenic iPSC models
55	Duchateau, 2024	NA	NA	NA	NA	*ABCA7* is identified as a risk gene for AD, with mutations linked to increased dementia risk. Its functions in lipid metabolism, phagocytosis, and amyloid deposition suggest a significant role in the pathogenesis of dementia
56	von Maydell, 2024	Matched control individuals	36 human *post mortem* samples	NA	Single‐nuclear RNA sequencing	*ABCA7* loss‐of‐function variants significantly increase AD risk by disrupting lipid metabolism, causing mitochondrial dysfunction, DNA damage, and NF‐kB signaling alterations in neurons, thereby contributing to the pathogenesis of dementia through these cellular disruptions
57	Duchateau et al., 2023	CSF analysis and *ABCA7* genotyping	229 AD patients	NA	*ABCA7* genotyping	*ABCA7* expansion carriers have lower Aβ1‐42 levels. Reduced inflammatory response
58	Bossaerts et al., 2022	Targeted sequencing	Cases: 1376 Belgian AD patients; control cohort: 976 Belgian individuals	NA	Targeted sequencing of *ABCA7* gene variants	Rare *ABCA7* missense mutations contribute to AD risk. Mutations cause protein mislocalization, reducing functional *ABCA7* at plasma membrane
59	Oblak, 2022	In vitro studies In vivo studies Human‐based studies	NA	NA	NA	*ABCA7* is a significant risk gene for late‐onset AD. *ABCA7* loss correlates with amyloid deposition and brain morphology
60	Gilmore, 2024	*APOE* genotype	180,345 participants	African ancestry	*APOE* genotyping	The prevalence of the *APOE* ε4 is notably higher among Black individuals, yet its association with dementia is weaker compared to Whites
61	Santos, 2024	*APOE* genotype	49 *post mortem* frontal cortex tissues	African ancestry	Quantitative characterization of local ancestry and *APOE* genotypes	The *APOE* gene, particularly the ε4 allele, increases AD risk. This study indicated that homozygous ε3 carriers of African ancestry exhibit lower *APOE* expression, potentially contributing to their reduced risk of developing dementia compared to those of European ancestry
62	Belloy, 2024	WGS	Participants aged 60+, European or admixed African ancestry	Admixed	Genetic data from SNP arrays or WGS	The paper identifies *APOE* ε4 as the strongest genetic risk factor for late‐onset AD, highlighting its interaction with a novel locus that may modulate its effects, particularly in populations of African ancestry, influencing dementia pathogenesis
63	Logue, 2011	Multi‐site studies, discordant sibling pairs, unrelated individuals	513 AD cases, 496 controls	African ancestry	Analyzed 2.5 million imputed genetic markers	The study found significant associations with SNPs in the *APOE* gene related to AD in African Americans, indicating its role in dementia pathogenesis. However, the causal variants may differ from those identified in White populations
64	Teruel et al., 2011	Nested case–control study	2928 residents aged 65 and over	African ancestry	NA	The *APOE* gene, particularly the *APOE* ε4 allele, is associated with dementia risk. In the study, its effect varied by ethnic identity and African admixture, suggesting complex interactions influencing dementia prevalence among African and admixed populations
65	Lwere, 2024	Recruited from two villages in Wakiso district	43 participants aged ≥ 65 years	Africans	*APOE* genotyping	*APOE* genotypes varied; ԑ3/ԑ3 most common in Ugandans. Low *APOE* ԑ4 frequency linked to lower AD risk.
66	Yoro‐Zohoun et al., 2021	Multicenter population‐based study design	Sample size estimated at ≈ 500 participants per site	Africans	*APOE* genotyping by PCR	*APOE* ε4 allele not significantly associated with neuropsychiatric symptoms. Protective trend observed in some models, needs further research
67	Raya, 2024	NA	1857 individuals from *post mortem* brain tissues	Admixed populations	NA	Women have higher dementia prevalence and CERAD scores. Black race affects CERAD scores and cognitive outcomes
68	Osuntokun, 1995	Case–control	56 subjects (39 controls, 17 dementia patients)	Africans	*APOE* genotyping	No association between *APOE* ε4 allele and AD in Nigerians. Higher ε4 frequency in controls than in AD patients
69	Stewart, 2001	Community‐based sampling from seven practices	202 participants aged 55–75 years	African ancestry	NA	The *APOE* gene, particularly the ε4 allele, is associated with increased cognitive impairment in African Caribbean populations, influenced by age and vascular risk factors like hypertension and diabetes, suggesting different pathways of effect compared to other populations
70	Rasmussen, 2020	Sequencing and genotyping methods used for analysis	105,597 individuals studied	NA	Sequencing and genotyping	Genetically low apoE levels increase dementia risk. High apoE levels decrease dementia risk
71	Paradela, 2023	Autopsy study in an admixed sample	648 participants	Admixed population	NA	*APOE* ε4 influences cognition via AD pathology mediation. Non‐AD pathologies did not mediate cognitive function
72	Ehn et al., 2025	Henome sequencing	Mother and daughter pair	NA	Genome sequencing	A complex genomic rearrangement causing *APP* triplication linked to familial autosomal dominant early onset AD, but it does not specifically address the role of the *APP* gene in dementia pathogenesis among Africans
73	El Kadmiri et al., 2014b	Clinical interviews and genetic sequencing	25 patients (17 sporadic, 8 familial cases)	African ancestry	Genetic sequencing	Mutations in the *APP* gene are linked to familial AD, contributing to Aβ accumulation, a key factor in dementia pathogenesis. This study identified novel mutations in Moroccan patients, highlighting genetic factors influencing AD in African populations
74	Athan, 2002	Community study	1013 elderly individuals	NA	PCR amplification	The paper identified polymorphisms in the *APP* promoter associated with AD susceptibility, particularly in African American patients. However, it concludes that these polymorphisms are unlikely to significantly influence *APP* expression or contribute strongly to dementia pathogenesis
75	Liu, 2022	Clinical data collection from patients	147 patients with *APP* mutations	NA	Analyzed genotype‐phenotype correlations based on clinical characteristics	APP mutations show typical AD clinical phenotypes. in onset age and behavioral symptoms identified
76	Wu, 2015	Case–‐control; cross‐sectional study	374 cases; 497 controls	NA	Environmental studies	The highest tertile of PM10 and ozone exposure, compared to the lowest, was linked to a greater risk of AD
77	Graves, 1998	Cross‐sectional study	89 AD and 89 controls	NA	Environmental studies	Exposure to aluminum at any point was linked to an increased risk of AD
78	Peters, 2013	Retrospective cohort study	1894 ever underground gold miners	NA	Environmental studies	Inhalation of aluminum dust was linked to a higher risk of AD‐related mortality
79	Schüz, 2009	Prospective cohort study	420,095 private mobile phone subscribers	NA	Environmental studies	Using a mobile phone was linked to a lower risk of hospitalization for AD
80	Muyela, 2023	Opportunistic household survey by Community Health Workers	672 older adults screened for dementia	Africans	Environmental studies	31.1% of older adults screened positive for dementia. Older age and illiteracy increase dementia screening positivity
81	Crane, 2023	Multi‐site cohort from Cardiovascular Health Cognition Study	2770 older adults	NA	Environmental studies	Higher toxic emissions linked to increased dementia odds
82	Park, 2024	NA	NA	NA	Environmental studies	Air pollution linked to increased dementia risk and severity
83	Tianyi, 2019	Random recruitment during house‐to‐house survey	501 individuals aged 50 years or older	Africans	Environmental studies	33.3% prevalence of cognitive impairment in rural elderly. Associated factors: age, sex, education, marital status, blood pressure
84	Pilleron et al., 2015	Urban areas used random sampling; rural areas used door‐to‐door approach	Minimum sample size was 500 participants per site	Africans	Environmental studies	Alcohol consumption reduces dementia probability in Central African Republic
85	Njamnshi, 2024	Community‐based approach for recruitment	103 participants aged ≥ 60 years	Africans	Environmental studies	10.7% prevalence of dementia in rural elderly. Onchocerca volvulus infection linked to cognitive decline
86	Heward et al., 2018	Community‐dwelling individuals from six villages	327 participants aged 65 and over	Africans	Environmental studies	High rates of cognitive decline in rural Tanzania. Education, grip strength, sex, and depression linked to decline
87	Awuol, 2022	Cross‐sectional study using quantitative methods	267 older adults interviewed	Africans	Environmental studies	46.2% prevalence of dementia among older adults studied. Low knowledge of dementia; only 8% had optimal understanding
88	Killin et al., 2016	Systematic review of published studies	60 studies included in the review	NA	Environmental studies	Moderate evidence for air pollution and aluminum as dementia risk factors. Need for more robust, longitudinal studies on environmental exposures
89	Okyere et al., 2024	Cross‐sectional design	384 participants aged 60 years or more	Africans	Environmental studies	Prevalence of dementia in Ghana is 16%. Age, education, and employment are significant risk factors
90	Samba, 2016	Longitudinal population‐based cohort study design	1029 participants at baseline	Africans	Environmental studies	Dementia increases mortality risk among older Congolese adults. Clinical severity and age are significant mortality predictors
91	Mfene, 2024	Cross‐sectional, one‐phased household study conducted over 8 months. Semi‐structured questionnaire and various dementia assessment tools administered	320 participants aged ≥ 60 years	NA	Environmental studies	Dementia prevalence was 13.4% in the sample. Age, education, and depression are significant risk factors
92	Awuol et al., 2023	Cross‐sectional study using quantitative methods	271 adults aged 50 and over	Africans	Environmental studies	46.2% prevalence of probable dementia among participants. Low knowledge of dementia; only 8% had optimal understanding
93	Ojagbemi, 2021	observational studies included in systematic review	8320 cohort risk years analyzed	Africans	Environmental studies	Low educational attainment is a key modifiable risk factor
94	Ayelagbe, 2011	Case‐control study design	100 elderly Nigerians (40 VD, 20 AD, 40 controls)	Africans	Environmental studies	Low omega‐3 fatty acids linked to dementia occurrence. Decreased plasma selenium associated with vascular dementia and AD
95	Guerchet, 2012	Cross‐sectional surveys in two cities	977 elderly Africans included in analysis	Africans	Environmental studies	Stressful life events impact dementia risk in low‐income countries
96	Yenesew, 2024	Screening of 445 publications for relevant studies	NA	NA	Environmental studies	Lower income and education levels are significant risk factors for dementia, with studies showing that individuals with less education are more susceptible to cognitive decline
97	Tyas, 2001	Prospective longitudinal study	694 cognitively‐intact older adults	NA	Environmental studies	Exposure to radiation in the workplace was linked to a higher risk of developing AD
98	Koeman, 2015	Case‐cohort study	Over a 17‐year follow‐up period, death certificates recorded 682 men and 870 women who had passed away without VD	NA	Environmental studies	In men, low or high exposure to extremely low frequency magnetic fields, compared to no exposure, was not linked to a higher risk of non‐vascular dementia mortality, whereas in women, no such pattern was observed. However, an association was found between electrical shocks and non‐vascular dementia mortality in women
99	Emard, 1994	Cross‐sectional study	129 individuals with AD	NA	Environmental studies	Fifteen individuals with AD were born in regions with below‐average lead levels, while 49 were born in areas where lead concentrations were above average
100	Feter et al., 2024	Population‐based survey across six countries	19,278 adults aged 50 years or more			37.6% of dementia cases are potentially preventable by modifiable risk factors. Less education, smoking, and inactivity have the highest attributable fractions
101	Chang, 2014	Retrospective cohort study	29537 (NO_2_ 29547) men and women of whom 1718 (1720) dementia cases	NA	Environmental studies	NO_2_ exposure revealed an increased risk of developing dementia.

Abbreviations: Aβ, amyloid beta; *ABCA7*, ATP‐binding cassette subfamily A member 7; AD, Alzheimer's disease; ADRD, Alzheimer's disease and related dementias; *AKAP9*, A‐kinase anchor protein 9; *APOE*, apolipoprotein E; BDNF, brain‐derived neurotrophic factor; CERAD, Consortium to Establish a Registry for Alzheimer's Disease; CRISPR, clustered regularly interspaced short palindromic repeats; CSF, cerebrospinal fluid; GWAS, genome‐wide association study; iPSC, induced pluripotent stem cell; NA, not applicable; NO_2_, nitrogen dioxide; PCR, polymerase chain reaction; *PSEN1*, presenilin 1; *PSEN2*, presenilin 2; qRT, quantitative reverse transcription; RT, real‐time; scRNAseq, single‐cell RNA sequencing; SNP, single nucleotide polymorphism; *TREM2*, triggering receptor expressed on myeloid cells 2; VD, vascular dementia; WES, whole‐exome sequencing; WGS, whole‐genome sequencing.

## 3 RESULTS AND DISCUSSION

### 3.1 Genetic risk factors

#### 3.1.1 APOE

##### Structure and isoforms of *APOE*

**FIGURE 2 alz70220-fig-0002:**
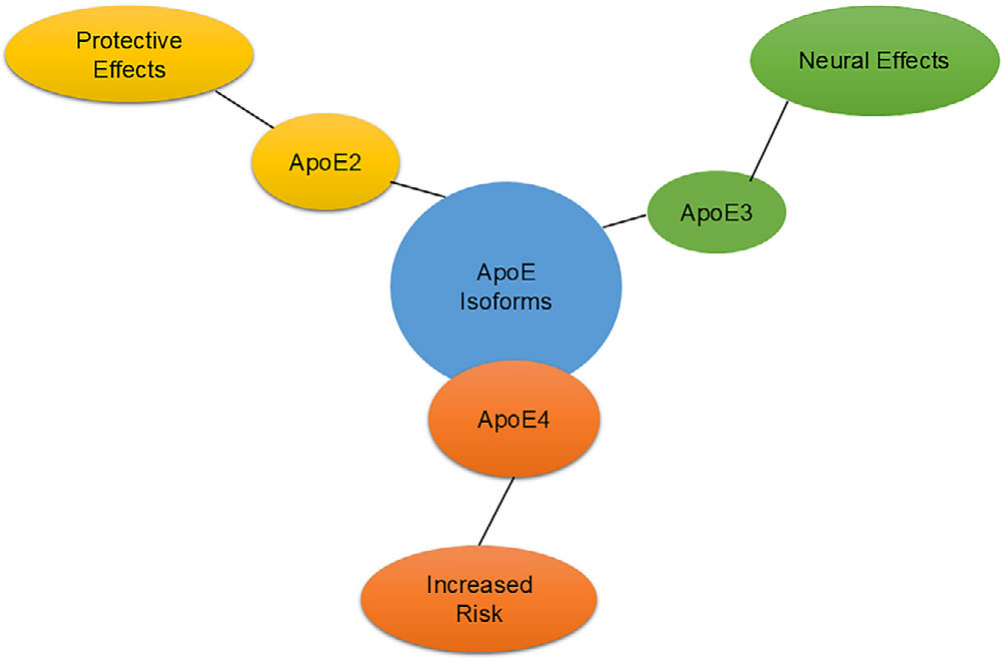
Visual summary of the three apoE isoforms (apoE2, apoE3, and apoE4) and their respective roles in dementia risk. apoE2 is protective, apoE3 is neutral, and apoE4 is associated with an increased risk of dementia. apoE, apolipoprotein E.

The *APOE* gene, located on chromosome 19, encodes a 299 amino acid glycoprotein.^30^ This gene is highly polymorphic, with three main isoforms (Figure 2): apoE2 (Cys112/Cys158), apoE3 (Cys112/Arg158), and apoE4 (Arg112/Arg158), corresponding to the ε2, ε3, and ε4 alleles, respectively.^31^, ^32^ These isoforms differ in their amino acid sequences, influencing their roles in cholesterol and phospholipid binding, brain homeostasis, neuroinflammation, and association with various diseases.^33^, ^34^ apoE3 is considered the most common and “normal” form.^35^ The alleles are defined by two single nucleotide polymorphisms (SNPs): rs429358 and rs7412.^36^ Inheritance of one allele from each parent results in nine common *APOE* genotypes: APOEε2/ε2, APOEε2/ε3, APOEε2/ε4, APOEε3/ε2, APOEε3/ε3, APOEε3/ε4, APOEε4/ε2, APOEε4/ε3, APOEε4/ε4; however, APOEε2/ε3, APOEε2/ε4, and APOEε3/ε4 are rare.^37^ While *APOE* ε4 is a major dementia risk factor, its effect appears less pronounced in African‐ancestry populations compared to European populations.^38^, ^39^, ^40^ For example, a study among elderly individuals in Nyeri, Kenya, found no significant association between *APOE* ε4 genotypes and dementia status.^41^ This highlights the need for further research in Africa to investigate the role of *APOE* ε4 in dementia risk and its underlying mechanisms.

##### Sources and physiological functions of apoE in the central nervous system

apoE is synthesized and secreted by various tissues and cells, including macrophages.^42^ The liver is the primary source of apoE, which can be found on cell surfaces or within secretory vesicles.^35^ The brain is the second largest producer of apoE,^42^, ^43^ primarily by astrocytes within the central nervous system (CNS).^44^, ^45^ Specialized astrocytic cell types, such as Bergmann glia of the cerebellum and pituicytes of the neurohypophysis, also synthesize apoE.^35^, ^43^ While typically produced by astrocytes, neurons can synthesize apoE under neuropathological conditions, often associated with the *APOE* ε4 isoform, which is linked to several neurodegenerative diseases. apoE is also present in the glia surrounding motor and sensory neurons in the peripheral nervous system and in non‐myelinating Schwann cells. Astrocytes secrete apoE as part of high‐density lipoproteins (HDLs), crucial for lipid transport and cholesterol metabolism. Furthermore, apoE is involved in amyloid precursor protein processing and the production, deposition, and clearance of Aβ.^42^

##### apoE and lipid metabolism in the CNS

In the CNS, cholesterol biosynthesis is primarily carried out by glial cells, including astrocytes, oligodendrocytes, and microglia, with neurons contributing to a lesser extent. apoE is the principal cholesterol carrier and major lipoprotein found on HDL‐like particles in the brain, exhibiting a high affinity for cell surface lipoprotein receptors and acting as the main ligand for the low‐density lipoprotein (LDL) receptor and LDL receptor–related protein.^42^

apoE plays a crucial role in brain cholesterol and lipid metabolism.^43^, ^46^ CNS lipoproteins are vital for redistributing cholesterol and lipids to neurons and other brain cells, supporting neuronal plasticity by facilitating membrane repair, organelle biogenesis, and synaptogenesis. apoE contributes to this process by forming apoE‐enriched CNS lipoproteins, resembling plasma HDL but differing from the primary ApoAI‐enriched HDL found in plasma.^43^

The brain uses a distinct cholesterol turnover process: excess sterols are converted to 24S‐hydroxycholesterol, a more soluble metabolite believed to cross the blood–brain barrier for excretion via the liver through bile, as cholesterol does not directly leave the brain.^43^

apoE is the predominant apolipoprotein in cerebrospinal fluid (CSF), exhibiting higher sialylation levels in CSF than in plasma.^44^ Plasma apoE levels vary by genotype, with *APOE* ε2 carriers exhibiting the highest and *APOE* ε4 carriers the lowest. These differences are partially due to apoE2's reduced LDL receptor binding, increasing plasma levels, and apoE4's preference for very‐low‐density lipoprotein, accelerating hepatic clearance and resulting in lower levels. In the brain, higher apoE2 levels may result from impaired receptor binding, while lower apoE4 levels likely stem from its unique structure. apoE4 is less stable than apoE3 due to an interaction between its amino and carboxyl ends, forming a “molten globule” intermediate.^43^, ^47^ This structural feature increases degradation, particularly in astrocytes, reducing its availability for cholesterol transport. These lower apoE4 levels may negatively impact cholesterol homeostasis and neuronal plasticity, both essential for brain function.

##### apoE4 and the risk of dementia

While neurons can produce apoE, they typically do so at low levels unless stressed or injured by factors such as aging, neurotoxins, ischemia, oxidative stress, or other environmental and genetic influences, conditions that necessitate cholesterol and lipid redistribution for repair.^44^, ^48^, ^49^ However, due to domain interaction, apoE4 is more susceptible to degradation by a neuron‐specific protease compared to apoE3.^43^, ^50^, ^51^, ^52^ This degradation generates neurotoxic carboxyl‐terminal fragments that escape the secretory pathway, promote tau phosphorylation, and disrupt mitochondrial function. Patients with dementia, particularly AD, often carry at least one ε4 allele and typically exhibit greater amyloid deposition than those without the ε4 allele.^53^

Although *APOE* ε4 was traditionally thought to primarily influence Aβ buildup leading to cognitive decline, recent research indicates that apoE affects other disease processes beyond Aβ accumulation.^32^ apoE is also implicated in neurotoxicity, blood–brain barrier permeability, mitochondrial dysfunction, and tau phosphorylation in AD.^35^, ^54^

The apoE isoforms differentially impact dementia risk due to their structural differences and distinct effects on Aβ. apoE4 is associated with a greater plaque burden compared to apoE3, while apoE2 is considered protective.^55^, ^56^ apoE4 plays a significant role in the early stages of Aβ plaque development, promoting increased formation and accumulation of plaque deposits in astrocytes.^35^ apoE4 prolongs the half‐life of Aβ and inhibits its clearance from the brain,^56^, ^57^ as well as its enzymatic degradation.^58^

Besides plaque formation, apoE facilitates Aβ clearance through receptor‐mediated clearance and proteolytic degradation. Aβ and apoE4 bind to the low‐density lipoprotein receptor‐related protein 1 receptor to facilitate Aβ protein.^59^ However, this clearance is impaired in *APOE* ε4 carriers due to the weaker binding between apoE4 and Aβ.^60^ apoE4's competitive binding behavior increases its affinity for Aβ receptor sites, significantly impairing Aβ clearance in *APOE* ε4 carriers. Because apoE4 transports lipids less efficiently than apoE2 and apoE3, Aβ clearance is also reduced.^61^

**FIGURE 3:**
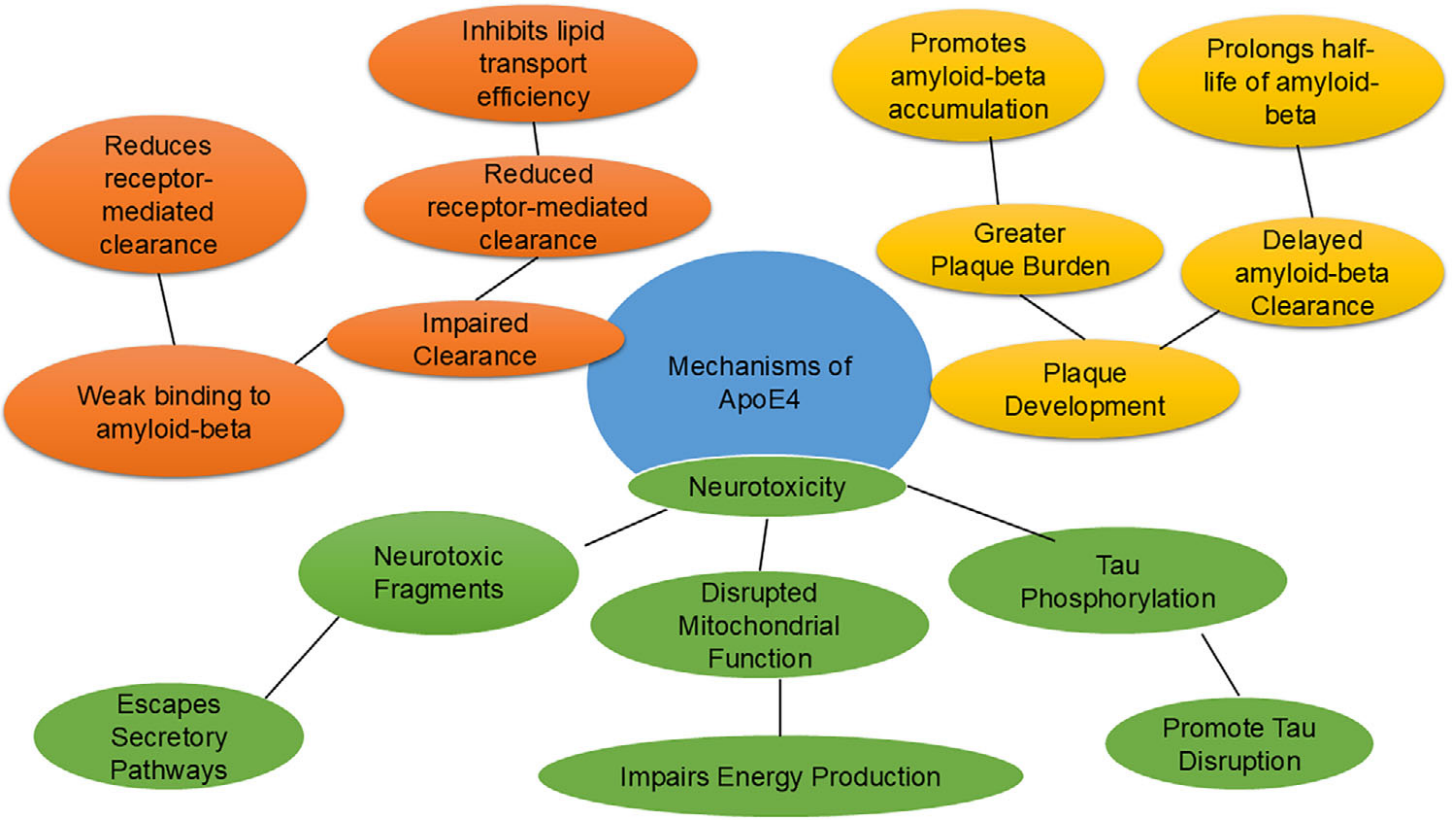
An overview of the mechanisms by which apoE4 contributes to dementia. This includes its role in plaque development, neurotoxicity (e.g., tau phosphorylation and mitochondrial dysfunction), and impaired clearance of Aβ due to weak binding and inefficient lipid transport. Aβ, amyloid beta; apoE, apolipoprotein E.

apoE plays a central role in dementia, particularly AD, by co‐depositing with Aβ in amyloid plaques.^53^, ^62^ Numerous studies have identified apoE4 as a significant risk factor for dementia‐related AD in various populations.^63^, ^64^, ^65^ However, in a population‐based study on the prevalence and incidence of AD and dementia in Yoruba individuals aged ≥ 65, it was reported that, despite the significant correlation between *APOE* ε4 homozygosity and AD in the Yoruba population, the association between the ε4 allele and AD seemed to be weaker in Yoruba individuals than in African American cohorts.^66^ These mechanisms have been shown in[Fig alz70220-fig-0002] Figure 3.

##### Quantification of total apoE and its isoforms in CSF

For analysis, isoform‐specific tryptic peptides corresponding to ε2, ε4, APOEε2/ε3, and APOEε3/ε4 are used, along with two peptides common to all three isoforms. Prior to sample preparation, stable isotopes are labeled with both ^13^C and ^15^N at the C‐terminal arginine (resulting in a +10 Da mass shift). Internal standard (IS) peptides are spiked into CSF samples at a final concentration of 0.067 µmol/L.^67^

Sample preparation begins with reducing 20 µL of CSF and 25 µL of the apoE IS mix using 30 mM dithiothreitol in ammonium bicarbonate at 60°C for 30 minutes.^63^, ^64^, ^65^ Alkylation is then performed using 70 mM iodoacetamide in ammonium bicarbonate, incubated at room temperature in the dark for 30 minutes. Digestion is carried out at 37°C for 2 hours using a trypsin/Lys‐C mix (20 µg/mL final concentration in 50 mM ammonium bicarbonate). The reaction is quenched with 10% trifluoroacetic acid. Solid‐phase extraction (SPE) is performed using hydrophilic–lipophilic balance (HLB) 96‐well µElution plates with minor modifications to the original protocol. Samples are washed with water, eluted with 100% methanol, and dried by vacuum centrifugation. Processed samples are stored at −80°C until liquid chromatography–mass spectrometry (LC‐MS) analysis. Quality control is maintained using three pooled CSF samples evenly distributed across the 23 96‐well plates used for analysis.^67^

Before LC‐MS analysis, samples are reconstituted in 100 µL of 50 mM ammonium bicarbonate. A 50 µL aliquot is then loaded onto a reversed‐phase high‐performance liquid chromatography C18 column (1.9 µm particle size, 2.1 mm internal diameter, 100 mm length). Peptide separation is achieved using gradient elution with mobile phase A (0.1% formic acid in water) and mobile phase B (0.1% formic acid in 84% acetonitrile and water). The flow rate is maintained at 300 µL/minute, with a gradient from 0% to 30% B over 5.5 minutes, resulting in a 10 minute total cycle time. Parallel reaction monitoring (PRM) is performed using a high‐resolution hybrid quadrupole‐orbitrap mass spectrometer with electrospray ionization. Instrument settings include an automatic gain control target of 3 × 10⁶ and a maximum injection time of 125 ms. Fragment spectra are acquired via scheduled PRM within 30 second retention time windows for each peptide. The isolation window is set at *m/z* ^3^, with separate acquisitions for endogenous and IS peptides. Collision energies are manually optimized for each peptide.

Other methods for *APOE* gene analysis include immunoturbidimetric assay, surface plasmon resonance (SPR), fiber optic particle plasmon resonance (FOPPR), and voltammetric detection. SPR quantifies the *APOE* gene and differentiates genotypes without polymerase chain reaction (PCR) amplification. This label‐free method uses DNA sequence hybridization to biotinylated probes, with restriction enzyme cleavage indicating specific sequences. It is simple, highly sensitive, and can detect as low as 10 fM of complementary sequences.^68^ The immunoturbidimetric assay (e4Risk test) is a latex‐enhanced assay for *APOE* ε4 detection in plasma, offering high diagnostic accuracy (99%) comparable to PCR, with advantages in automation, cost, and throughput. This method is adaptable to clinical chemistry analyzers, allowing for integration into routine diagnostics.^69^ FOPPR biosensors, combined with ligase reactions, enable DNA amplification‐free *APOE* genotyping. This method uses gold‐iron oxide nanoparticles and biotinylated probes for high sensitivity and specificity, with detection limits ranging from 16 fM to 38 fM and results consistent with DNA sequencing.^70^ Voltammetric detection uses ferrocene‐capped gold nanoparticles for signal amplification in detecting the *APOE* ε4 gene. This method involves enzymatic cleavage of specific sequences and has a detection limit of 0.1 pM. Validated with genomic DNA samples, it demonstrates the potential for early AD diagnosis.^67^

#### 3.1.2 ABCA7

##### Site of synthesis, structure, and function

*ABCA7*, closely related to *ABCA1*, is a phospholipid transporter involved in cholesterol efflux.^71^, ^72^ Highly expressed in the brain, particularly in microglia,^73^ *ABCA7* has been identified as a risk factor for AD in genome‐wide association studies.^74^, ^75^ Microglia exhibit the highest *ABCA7* expression among brain cells.^76^ *ABCA7* is also expressed in other tissues and organs, including the spleen and lungs.^62^

*ABCA7* belongs to the ABC transporter gene superfamily and is a member of the A subfamily. The *ABCA7* gene encodes a 2146‐amino acid transmembrane transporter protein with a molecular weight of 220 kDa.^53^ *ABCA7* plays a significant role in transporting and distributing lipids and other lipophilic molecules. Specifically, it is involved in lipid transport and phagocytosis, processes crucial for amyloid clearance. *ABCA7* facilitates the clearance of Aβ peptides, and its deficiency has been linked to increased cognitive decline.^72^, ^77^

##### *ABCA7* gene and risk of dementia

*ABCA7* regulates cholesterol efflux and phospholipid transport, crucial for neuronal membrane integrity and the function of apolipoproteins like apoE.^78^ It is also involved in lipid metabolism and immune responses, processes relevant to neurodegenerative diseases.^79^, ^80^ Mutations in *ABCA7* that affect lipid transport can lead to increased amyloidogenic processing and deficient Aβ clearance. *ABCA7* mutations are found in ≈ 7% of AD patients, with specific variants linked to increased dementia risk.^77^ Loss‐of‐function variants can lead to mitochondrial dysregulation, affecting neuronal function.^81^ These loss‐of‐function variants, associated with increased dementia risk, particularly AD type, suggest that *ABCA7* deficiency contributes to dementia by altering mitochondrial lipid metabolism and impacting neuronal function. This review identified five *ABCA7* SNPs (rs7247601, rs3752231, rs4147914, rs4147937, and rs2074453) associated with AD, highlighting the gene's potential role in dementia risk.^82^

Several studies have emphasized that SNPs in the *ABCA7* gene significantly increase the genetic risk for AD‐type dementia in African Americans. However, these studies often do not investigate the potential dementia risk among African populations or provide comparative data to illuminate differences between these groups. Most studies have focused on the association between *ABCA7* and AD risk in African Americans, reporting stronger associations compared to non‐Hispanic White adults.^23^, ^83^, ^84^ In non‐Hispanic individuals, *APOE* ε4 is the strongest genetic risk factor for AD, contributing to a 20% to 50% increased risk.^85^ Notably, very few, if any, studies have been identified within African populations themselves.

##### APP, PSEN1, PSEN2, and TREM2 genes

The *APP*, *PSEN1*, *PSEN2*, and *TREM2* genes are involved in key physiological processes, including synaptic plasticity, neuronal differentiation, and the regulation of Aβ protein production. Mutations in *APP*, *PSEN1*, and *PSEN2* directly disrupt *APP* processing, resulting in increased Aβ production, a hallmark of AD.

APP, a membrane‐associated protein with a single transmembrane domain, may function as a receptor.^19^ The predominant isoform in the CNS consists of 695 amino acids.^19^, ^86^, ^87^, ^88^ *APP* undergoes sequential proteolytic cleavage by either α‐ or β‐secretase (also known as beta‐site *APP* cleaving enzyme [BACE]), followed by γ‐secretase.^89^ A recently discovered third pathway, involving η‐secretase, requires further investigation to determine its functional significance.^90^

**FIGURE 4:**
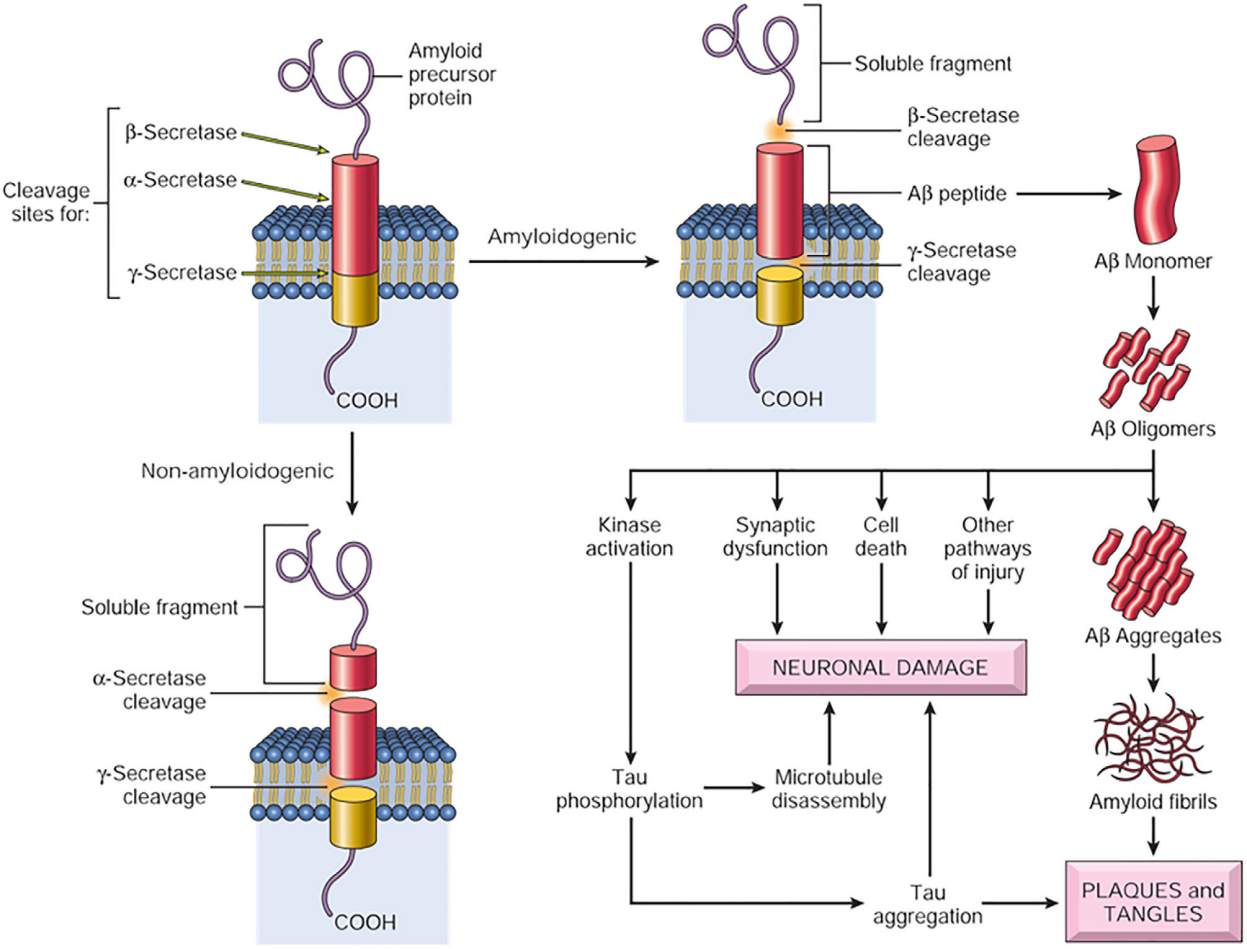
An illustration of protein aggregation in AD, which involves the cleavage of APP. When APP is processed by α‐secretase and γ‐secretase, it generates a benign soluble peptide. In contrast, cleavage by beta‐site APP cleaving enzyme (BACE) and γ‐secretase produces Aβ peptides, which aggregate to form harmful structures. These aggregates are central to the development of the plaques and tangles characteristic of AD. Retrieved from Kumar, V., Abbas, A. K., Fausto, N., & Aster, J. C. (2021). Robbins and Cotran pathologic basis of disease, 9th edition, page 1277. Aβ, amyloid beta; AD, Alzheimer's disease; APP, amyloid precursor protein.

Cleavage by α‐secretase initiates the non‐amyloidogenic pathway, generating the *APP* intracellular domain (AICD) and the soluble, extracellular fragment APPsα. APPsα mediates most of the recognized neuroprotective and neurotrophic effects attributed to *APP*. Conversely, cleavage by β‐secretase initiates the amyloidogenic pathway, producing AICD, a secreted APPsβ fragment, and crucially, Aβ peptides ranging from 38 to 43 amino acids in length (Figure 4). Aβ exists in various monomeric or multimeric soluble forms and can aggregate into fibrils and plaques. Aβ42, though less abundant than Aβ40, has a greater tendency to aggregate, driving plaque formation. The extracellular accumulation of amyloid plaques, along with the intracellular deposition of tau fibrils, is the defining histopathological characteristic of AD.

Some *APP* mutations are located near the β‐secretase and γ‐secretase cleavage sites, while others reside within the Aβ sequence itself, increasing its aggregation propensity. The two genes primarily responsible for early‐onset familial AD encode the presenilin proteins: *PSEN1* (chromosome 14) and *PSEN2* (chromosome 1). Mutations in these genes result in a gain of function, causing the γ‐secretase complex to produce increased levels of Aβ, especially the more aggregation‐prone Aβ42 isoform.

#### 3.1.3 Novel findings and population‐specific variants

A key finding of this review is the identification of novel genes and rare variants in African ancestry which are not widely studied in other ethnic groups. Studies by Sherva et al. and Logue et al. identified rare variants in genes like *AKAP9* and cytidine deaminase (*CDA*) that were either unique to or had a stronger association with AD in African‐descent populations.^91^, ^92^

The discovery of these novel genes, such as *AKAP9*, highlights the genetic diversity within African populations and underscores the need for further research focused on these understudied groups to uncover previously unexplored genetic risk factors for dementia. These rare variants suggest that targeted sequencing in African populations may reveal significant insights into dementia risk currently missing from large‐scale genome‐wide association studies focused primarily on European populations.

*AKAP9* has emerged as a significant factor in AD risk, particularly among African Americans. Recent studies have identified rare *AKAP9* mutations, such as rs144662445 and rs149979685, associated with increased tau phosphorylation and altered tau interactome, key processes in AD pathology.^93^ *AKAP9* is a binding protein that anchors protein kinase A (PKA) to specific intracellular sites, enhancing its catalytic efficiency by increasing sensitivity to cyclic adenosine monophosphate (cAMP).^94^, ^95^ *AKAP9* can also recruit adenylyl cyclase to produce the cAMP necessary for PKA activation.^96^ PKA promotes tau phosphorylation by enhancing the activity of glycogen synthase kinase‐3β (GSK‐3β).^97^ Individuals carrying one or both of these *AKAP9* variants had a 2.75‐fold higher risk of developing AD.^98^ These SNPs were absent in > 4000 sequenced individuals of European ancestry,^99^ indicating their specificity to individuals of African ancestry. These findings suggest a role for *AKAP9* in the molecular mechanisms underlying dementia risk.

The *CDA* gene has also been implicated in dementia risk, particularly through its role in neuroinflammatory processes and genetic susceptibility. *CDA* was identified as 1 of 93 significant risk genes for AD dementia through a Bayesian genome‐wide transcriptome‐wide association study (TWAS) integrating cis‐ and trans‐expression quantitative trait loci data from brain and blood tissues.^100^ Alterations in *CDA* may influence immune responses, which play a crucial role in the development and progression of neurodegenerative diseases like AD.^101^

##### Prevalence of genetic risk factors for dementia in African‐ancestry populations

Recent studies have identified several genetic variants associated with dementia risk in African populations, with some showing significant differences compared to Western‐ancestry populations. The *APOE* ε4 allele, a well‐established risk factor for AD in Western populations, has a complex and less pronounced effect in African populations. Studies in sub‐Saharan Africa indicate that while the *APOE* ε4 allele is present at comparable or even higher frequencies than in Western populations, its association with AD risk appears weaker.^38^, ^39^, ^40^ For example, in a population‐based study on the prevalence and incidence of AD and dementia in Yoruba individuals aged ≥ 65, it was reported that, despite the significant correlation between *APOE* ε4 homozygosity and AD in the Yoruba population, the association between the ε4 allele and AD seemed to be weaker in Yoruba individuals than in African American cohorts.^102^ This suggests that additional genetic or environmental factors may modify the impact of *APOE* ε4 in African populations.

Beyond *APOE*, the *ABCA7* gene has emerged as a risk factor in African ancestry. Genetic association studies have shown that loss‐of‐function variants in *ABCA7* significantly increase AD risk among African Americans, with effect sizes larger than those reported in European populations.^84^ However, data on *ABCA7* in native African populations remain scarce, underscoring the need for targeted studies to validate its role in dementia risk within Africa.

Additionally, rare genetic variants unique to African populations have been identified, including those in the *AKAP9* and *CDA* genes. These variants, largely absent in European populations, have been linked to increased tau phosphorylation and neuroinflammation, both of which are key mechanisms in dementia pathology.^92^ The identification of these population‐specific risk factors highlights the importance of expanding genomic studies to underrepresented populations to enhance our understanding of dementia susceptibility in diverse ancestries.

### 3.2 Environmental risk factors

#### 3.2.1 Environmental risk factors associated with dementia

Environmental factors, including high temperatures, socioeconomic status (SES), and air pollution, have been linked to increased dementia risk.^103^, ^104^, ^105^ Specifically, toxic chemical emissions from industrial facilities and exposure to ambient air pollutants like NO_2_ and PM2.5 are associated with a higher risk of dementia, particularly in the elderly.^106^, ^107^ Exposure to heat waves also increases dementia mortality, placing Africa at heightened risk.^108^, ^109^, ^110^

Sustained high body temperatures can impair cognitive function and exacerbate AD pathologies, as demonstrated in animal studies.^111^ Elevated temperatures increase Aβ peptide generation and tau phosphorylation, both critical in AD pathology, by increasing BACE1 levels and decreasing neprilysin, leading to amyloid pathology.^111^

In Ghana, studies show rising mean temperatures across major cities, with geographic and urban variations. Wa reports the highest mean temperature (30.76°C), while Accra and Kumasi report lower averages (27.86°C and 27.15°C, respectively).^112^ Historical data indicate a mean annual temperature increase of 1.0°C from 1961 to 2000, with projections of up to a 2°C increase by 2080 under high emission scenarios.^113^ The Upper East region is projected to experience a peak increase of 1.80°C by 2065.^114^ These temperature increases place the Ghanaian population at increased dementia risk.

The relationship between SES and dementia risk is complex; lower SES is associated with a significantly increased likelihood of developing dementia.^115^, ^116^ Individuals with lower SES often have limited access to quality education, health care, and resources that support healthy lifestyles, all of which offer protection against cognitive decline. Lower educational attainment, for example, is strongly associated with an increased risk of dementia‐related death, even after controlling for common risk behaviors and comorbidities.^117^

##### Potential mechanisms of interaction between genetic and environmental factors

**FIGURE 5:**
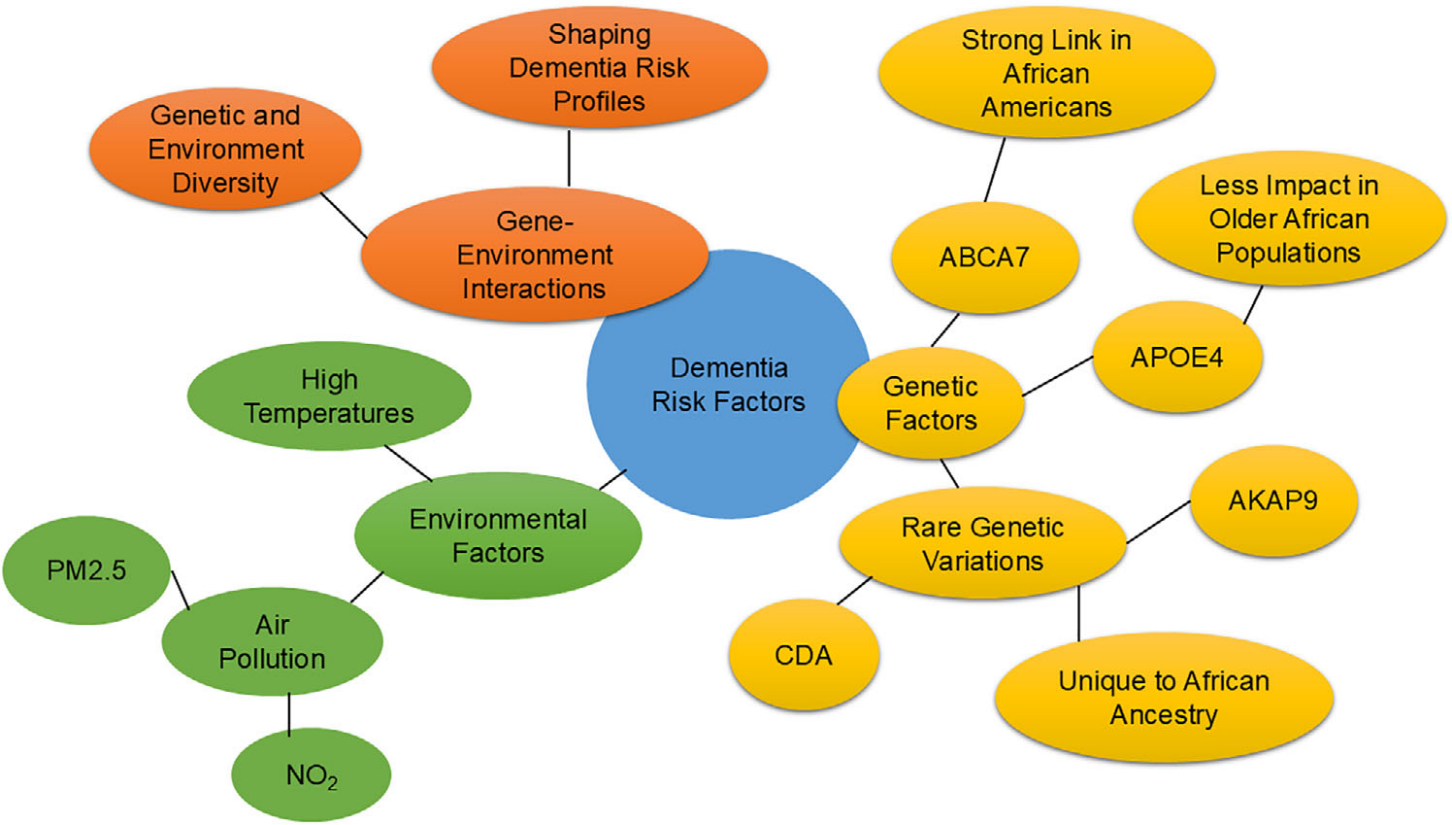
Visual summary of gene–environment interactions influencing dementia risk. The interactions between genetic diversity and environmental exposures highlight their combined impact on shaping dementia risk profiles in diverse populations. *ABCA7*, ATP‐binding cassette subfamily A member 7; *AKAP9*, A‐kinase anchor protein 9; *APOE*, apolipoprotein E; *CDA*, cytidine deaminase; NO_2_, nitrogen dioxide; PM2.5, particulate matter.

The development of dementia of any kind is influenced by a complex interaction between genetic and environmental factors. These interactions can be seen through a variety of mechanisms, which often involve biological, epigenetic, oxidative stress and mitochondrial dysfunction, and environmental factors. *APOE* ε4 is one of the most well‐known genetic risk factors for AD. However, the risk associated with *APOE* ε4 is influenced by environmental factors like air pollution, exercise, and cognitive engagement.^34^ Epigenetic mechanisms refer to changes in gene expression without altering the underlying DNA sequence. Environmental factors such as continuous exposure to heat, stress, and toxins can influence epigenetic changes and hormonal balance, and again can increase oxidative stress and neuroinflammation, which may exacerbate the effects of genetic susceptibility to dementia.^9^, ^10^, ^11^, ^12^ Genetic factors may determine susceptibility, while environmental factors can modify or exacerbate that risk. Understanding the precise mechanisms of these interactions is important for developing effective prevention strategies and treatments for dementia. These gene–environment interactions influencing dementia risk have been visually illustrated in Figure 5.

##### Barriers to dementia research in Africa

Dementia research in Africa faces significant barriers, including limited funding and resources. Many African countries prioritize infectious diseases over chronic conditions like dementia, resulting in underinvestment in dementia‐related research. There is a lack of comprehensive epidemiological data on dementia, making it difficult to understand its prevalence and risk factors in sub‐Saharan Africa.^4^, ^8^, ^118^ Misdiagnosis and underreporting of dementia cases further exacerbate this data gap. Additionally, genetic research on dementia in Africa is sparse, leaving a significant gap in understanding the genetic risk factors specific to the continent.^25^, ^26^, ^119^ Limited access to diagnostic tools, such as neuroimaging, hinders early detection and accurate diagnosis.^25^, ^26^, ^119^ There is also a shortage of health‐care professionals trained in dementia care and diagnosis, particularly in rural areas. Cultural stigma and misconceptions about dementia make it difficult for affected individuals to seek medical help and participate in research. The lack of specialized care facilities and social support systems for dementia patients further complicates research efforts. Finally, limited international collaboration and underrepresentation of sub‐Saharan Africa in global studies contribute to the slow progress in dementia research on the continent.^4^, ^8^, ^118^

## 4 CONCLUSION

Our review suggests that gene–environment interactions may significantly modify dementia risk in sub‐Saharan Africans. The weaker effect of *APOE* ε4 in African ancestry, despite its relatively high frequency, indicates that socioeconomic conditions and health‐care access may interact with genetic predispositions differently in sub‐Saharan Africans compared to other groups. The genetic variants like *ABCA7* and *AKAP9* have been predominantly studied in African American populations, but their validation in African cohorts remains limited. This highlights the urgent need for more extensive studies across diverse sub‐Saharan Africa to validate these findings. The findings from our review also highlight the need for public health education. Public health initiatives focused on dementia prevention and diagnosis in sub‐Saharan Africa must consider the genetic diversity and unique risk factors present. Furthermore, interventions developed based on studies in the global north populations may not fully address the genetic risks present in sub‐Saharan Africans.

To address the significant barriers to dementia research in Africa, several actionable recommendations are proposed. First, capacity building is essential to train health‐care professionals in dementia diagnosis, care, and research methodologies. This includes establishing specialized training programs and incorporating dementia education into the curriculum of medical schools and continuing professional education.

Second, investments in infrastructure and funding are critical to support local biobanks and research facilities. Biobanks that prioritize the genetic diversity of African populations can enable the identification of unique genetic risk factors, facilitating region‐specific diagnostic tools and therapies. Increased funding will also support the development of epidemiological studies to fill the existing data gaps on dementia prevalence and risk factors.

Third, region‐specific awareness campaigns are needed to combat cultural stigma and misconceptions about dementia, which often delay diagnosis and hinder research participation. Community‐based outreach programs can educate the public about dementia risk factors, symptoms, and the benefits of early detection and research participation.

Last, fostering international collaborations can provide African researchers access to advanced technologies, expertise, and global datasets, while ensuring the region's representation in global dementia studies. These partnerships can also facilitate the sharing of best practices and resources for dementia research and care.

By addressing these barriers through coordinated efforts, it is possible to close the research gaps, improve early detection and management, and ultimately reduce the burden of dementia in sub‐Saharan Africa.

## AUTHOR CONTRIBUTIONS

Conceptualization—David Mawutor Donkor, Esther Marfo, Ansumana Bockarie, and David Larbi Simpong; methodology and investigation—David Mawutor Donkor, Esther Marfo, and John Dogah; supervision—David Larbi Simpong; writing, original draft—David Mawutor Donkor, Esther Marfo, Edward Jenner Tettevi, Maxwell Hubert Antwi, George Nkrumah Osei, and David Larbi Simpong. All authors revised the manuscript critically for important intellectual content. All authors read and agreed with the final manuscript.

## CONFLICT OF INTEREST STATEMENT

The authors declare no conflicts of interest. Author disclosures are available in supporting information.

## Supporting information



Supporting Information

Supporting Information

## Data Availability

No data were used for the research described in the article.
