# Supplementary material for: Genetic and environmental risk factors for dementia in African adults: A systematic review
Source: Alzheimers Dement. 2025 Apr 28;21(4):e70220. doi: 10.1002/alz.70220 (PMC12035544; doi:10.1002/alz.70220)
Supplement: Supplementary file 2 — Supporting Information [file ALZ-21-e70220-s002.docx]

# Search Strategy for Systematic Review

## Database: PubMed

Search Terms:

1. ("Dementia"[MeSH Terms] OR "Cognitive Decline" OR "Alzheimer's Disease") AND ("Africa" OR "African Population" OR "Sub-Saharan Africa") NOT (review)
2. ("Dementia"[MeSH Terms] OR "Cognitive Decline" OR "Alzheimer's Disease") AND ("Environmental Factors" OR "Socioeconomic Status" OR "Pollution") AND ("Africa" OR "African Population" OR "Sub-Saharan Africa") NOT (review)

## Database: Cochrane Library

Search Terms:
1. (dementia OR "Alzheimer’s disease") AND ("APOE4" OR "ABCA7" OR "AKAP9" OR "CDA" OR "APP" OR "PSEN1" OR "PSEN2" OR "TREM2") AND ("African ancestry" OR "Sub-Saharan African population")

Genetic Risk Factors for Dementia in African based on Title, Abstract, Keyword

Filters Applied: None.

## Database: Google Scholar

Search Terms:
Dementia AND Genetics AND Environment AND Africa NOT Rview
